# A bivalent porcine circovirus type 2 (PCV2), PCV2a-PCV2b, vaccine offers biologically superior protection compared to monovalent PCV2 vaccines

**DOI:** 10.1186/s13567-022-01029-w

**Published:** 2022-02-18

**Authors:** Meggan Bandrick, Monica Balasch, Andrea Heinz, Lucas Taylor, Vickie King, Jeri Toepfer, Dennis Foss

**Affiliations:** 1 Zoetis Inc, Veterinary Medicine Research and Development, 333 Portage Street, Kalamazoo, MI 49007 USA; 2 Zoetis Manufacturing and Research Spain S.L. , Ctra. Camprodon s/n, Finca La Riba, 17813 Vall de Bianya, Spain

**Keywords:** PCV2, vaccine, evolution of virus strains/genotypes, cross-protection

## Abstract

Recent publications suggest PCV2 vaccine-induced protection is superior when the vaccine and challenge are closely matched. PCV2’s evolutionary rate, propensity for recombination, and genotype shifting, all provide rationale for modernizing PCV2 vaccines. One mechanism to increase a vaccine’s epitope breadth is by designing a bivalent vaccine. The objective of these studies was to evaluate efficacy of a monovalent (PCV1-2 chimera, cPCV2a or cPCV2b) and bivalent (cPCV2a–cPCV2b) vaccine in terms of homologous and heterologous efficacy. In Study A, pigs were vaccinated with cPCV2a or saline and challenged with PCV2a or PCV2b. In Study B, pigs were vaccinated with cPCV2a, cPCV2a–cPCV2b bivalent, or saline, and challenged with PCV2a. In Study C, pigs were vaccinated with cPCV2b, cPCV2a–cPCV2b bivalent, or saline, and challenged with PCV2b. In all studies vaccines and saline were administered intramuscularly to pigs at three to four weeks of age. Virulent PCV2b or PCV2a was administered to all animals approximately three weeks post-vaccination. Both mono and bivalent vaccinated groups demonstrated significantly lower viremia, percent of animals ever viremic, percent of animals with lymphoid depletion and/or histiocytic replacement, and percent of animals with PCV2 colonization of lymphoid tissues compared to saline controls. In Study A, a biologically relevant, though not significantly different, improvement in homologous versus heterologous protection was observed. In Studies B and C, biologically superior efficacy of the bivalent cPCV2a–cPCV2b vaccine compared to either monovalent vaccine was demonstrated. Taken together, cross-protection among mismatched PCV2 vaccine and challenge genotypes is not 100%; a bivalent PCV2 vaccine may provide the best opportunity to broaden coverage to circulating strains of PCV2.

## Introduction

Porcine Circovirus (PCV) is a small ~1700 bp, nonenveloped, single stranded DNA virus. PCV type 1 (PCV1) was identified in cell culture as a contaminant in the 1970s but has not been shown to cause disease in pigs. PCV type 2 (PCV2) has been known to affect pigs since 1969. PCV2 systemic disease has been considered a serious threat to the swine industry since 1985. There is evidence that it has been present in swine for over 100 years [1].

PCV2 is responsible for the previously called porcine multisystemic wasting syndrome (PMWS) also known as porcine circovirus type 2 systemic disease (PCV2-SD) and porcine circovirus type 2 subclinical infection (PCV2-SI). PCV2-SD refers to a range of clinical signs including post-weaning diarrhea, respiratory dyspnea, failure to gain weight, signs of anemia and icterus, and wasting disease whereby pigs fail to thrive. Other clinical signs including respiratory distress, tremors, enteric disease, dermatitis, nephropathy (PCV-NS), and reproductive failure (PCV-RD) are also seen in a condition now referred to as PCV-associated diseases (PCVAD) or more simply PCV diseases (PCVD). Hallmarks of severe PCV2 infection include high viremia levels, high virus titers within tissues, and lymphoid depletion/granulomatous inflammation. The severity of infection can be complicated by common co-infections with other viruses or bacteria. This, along with the potential need for a co-factor or disease inducing agent, has confounded attempts to fulfill Koch’s postulates using PCV2 alone. PCV2 disease does fulfill Evans’ postulates as a multifactorial disease. It is generally accepted that PCV2 is highly associated with PCVAD/PCVD. PCV2 associated diseases are now recognized as one of the most economically important global issues affecting growing swine with up to 20% mortality possible [2].

There are 8 proposed PCV2 genotypes labeled PCV2a-h [3]. PCV2 virus genotypes (a, b, d, etc.) are genetically similar containing two major structural proteins—the replicase encoded by ORF1 and the capsid encoded by ORF2. It is notable that this DNA virus has an evolutionary rate more like RNA viruses. Co-infection of PCV2 viruses is common, potentially leading to multiple PCV2 viruses interacting within a cell allowing further opportunities for genetic variation to occur [3–5]. Recombination occurs in 20–35% of PCV2 viruses and has been observed across most genotypes [3, 5].

Ongoing genetic change has led to molecular diversity within PCV2. Initial PCV2 infections were identified as PCV2a; however, since PCV2a was identified there have been two major genotype shifts resulting in a switch of predominant circulating virus from PCV2a to PCV2b with a later switch to PCV2d in many geographic locations worldwide [6–9]. PCV2c, e–h are detected less frequently. PCV2g and PCV2h are recently identified recombinant genogroups mostly comprised of gene segments from PCV2a, b, and d [3]. Genotypes a, b, and d are clinically relevant and most often associated with clinical PCV2-SD [3, 10]. New virus genotypes including recombinants are continually being sequenced with changes focused primarily on the capsid (ORF2) genetic sequence [11, 12].

Widespread use of PCV2a vaccines have greatly reduced the prevalence and severity of PCV2 viral infections. In fact, PCV2 vaccines represent a great success within the swine industry; PCV2 vaccines have historically successfully controlled PCV2 diseases. The success of PCV2a vaccines in reducing clinical disease to a variety of field strains is due in large part to common epitopes shared among a wide number of field genotypes and existing PCV2a vaccines. However, as minor changes in the capsid sequence accumulate, the shared epitopes among PCV2a-based vaccines and field strains wane. Even minor changes in the capsid amino acid sequence can alter the configuration of conformational epitopes and thereby affect PCV2 pathogenicity and immune differentiation including binding of neutralizing antibodies [13, 14]. A growing dissimilarity in epitopes shared among evolving field strains and constant vaccine strains may be contributing to decreasing efficacy of PCV2a vaccines [3, 12, 15–17]. The hypotheses that minor variations in epitopic regions can lead to differences in immune recognition [11, 15–17] and escape from the vaccine-induced immune response [11, 18] have been raised previously. Molecular evidence has documented the lack of complete cross-protection of PCV2 vaccines [18]. This is most likely explained by the genetic diversity among PCV2 virus capsid epitopes and is supported by vaccine lack of efficacy cases where the vaccine and field strain were mismatched [12, 19]. A summary of challenge outcomes in homologous and heterologous vaccine-challenge studies provides evidence that PCV2a vaccines induce cross-protection to divergent genotypes to some extent [18]. There is also evidence that vaccines based on the same genotype of the challenge strain are better at reducing viremia in challenge experiments [18]. Taken together, PCV2 vaccine protection to a variety of field virus genotypes can be attributed in part to the shared epitopic determinants among vaccine and field viruses.

PCV2a vaccines have successfully limited prevalence of PCV2a viruses, but likely supported increased diversity of epitopes within PCV2a viruses [20] and immune pressure to support evolution of newer genotypes [21]. Importantly, PCV2 vaccines do not provide sterilizing immunity [12, 15], allowing subclinical PCV2 infections, which remain a major economic burden [22], to continue in vaccinated herds with some vaccinated herds still reporting clinical disease. Furthermore, traditional single genotype PCV2 vaccines, while they do offer cross-protection, may not offer enough coverage to include evolving field viruses [12]. Taken together, the high genetic variability within PCV2 has resulted in PCV2a vaccines being less effective [1, 23] and there is a growing need to improve existing vaccines. An improved PCV2 vaccine, having high similarity in epitopes between vaccine and field PCV2 viruses, would reduce the potential for emerging PCV2 viruses that are pathogenic and not susceptible to current vaccine strategies, as well as improve protection to subclinical infections.

The hypothesis that a genotype-matched vaccine and challenge would provide better efficacy than a heterologous vaccine-challenge match was first investigated. Finding that cross-protection was incomplete and keeping in mind the inherent diversity within PCV2, it was hypothesized that a vaccine comprised of a combination of two PCV2 genotypes, PCV2a and PCV2b, would provide better protection than a monovalent vaccine in pigs challenged with either PCV2a or PCV2b.

## Materials and methods

Animals were housed and managed in strict accordance with the recommendations in the Guide for the Care and Use of Laboratory Animals of the National Institutes of Health. The protocols were approved by the Zoetis Institutional Animal Care and Use Committee (Protocol Number: KZ-1655e-2011-11-tkh), and by IACUC of the participating study sites.

### Studies A, B, and C

#### Animals

Commercial crossbred pigs were used; in each study, pigs originated from a single batch. Study B and C pigs were from the same source farm. Pigs were farrowed from sows with no recent history of vaccination against or clinical problems with PCV2. Male and female pigs approximately 3–4 weeks of age at Day 0 were sourced from farms with no recent history of disease with *Glaserella parasuis*, porcine reproductive and respiratory syndrome virus, *Mycoplasma hyopneumoniae*, swine influenza virus, or PCV2. Piglets were clinically healthy, seronegative to PCV2 by SERELISA® PCV2 Ab Mono Blocking enzyme linked immunosorbent assay (ELISA; performed per manufacturer instructions) on Day 0 (SERELISA S/Nc ratio > 0.5; the lower the S/Nc SERELISA value the more positive the value is and the higher the antibody level), and negative for PCV2 viremia by qPCR (performed as described [24]) on Day 0 (the positive/negative cutoff was determined for each assay and dependent on the assay performance and the positive control within each assay). All animals were randomized to treatment.

#### Allotment/randomization/replication

Pigs were allotted using a randomized complete block design produced by the Biometrics Representative using a SAS program [25]. The pigs were blocked by litter, with an equal number of pigs per block. Each piglet within a block originated from the same litter/sow. Blocks of pigs remained together and in their assigned pens throughout the entire length of the study. Pig was the experimental unit.

#### Masking

The investigator and individuals who performed observations, assays, and/or were involved in data collection were masked. The information of which treatment a group of animals received, or the treatment group to which the animal had been assigned was concealed to all masked individuals.

#### Vaccination

Experimental preparations of inactivated Porcine Circovirus Vaccine Type 1–Type 2a Chimera (cPCV2a) and Porcine Circovirus Vaccine Type 1–Type 2b Chimera (cPCV2b) were prepared as monovalents or as a bivalent vaccine. cPCV2a vaccine potency targets and cPCV2b vaccine potency targets were similar across studies. All vaccines were formulated with the same adjuvant and final adjuvant concentration, MetaStim®, an oil-in water emulsion. The experimental monovalent cPCV2a vaccine was used in the studies A and B; monovalent cPCV2b was used in the Study C, while the bivalent cPCV2a-cPCV2b vaccine was used in the studies B and C.

Pigs were approximately 3–4 weeks of age at the time of vaccination (Day 0 of the corresponding study). A single dose of 2 mL of the assigned vaccine was administered intramuscularly (IM) into the right neck by an unmasked treatment administrator. Physiologic saline was used as a negative control.

#### Challenge material

PCV2a (strain 40895; GenBank accession number AF264042) and PCV2b (strain NMB; GenBank Accession number GU799576) challenge material were originally isolated from pigs diagnosed with Postweaning Multisystemic Wasting Syndrome (PMWS). Challenge material were independently isolated and both from pigs located in Iowa, United States of America. Pathogenicity of these strains have been previously described [26, 27]. PCV2a and PCV2b challenge viruses were expanded on PK15 cells with final titers of approximately 5.4 ± 0.5 log_10_ and 6.1 ± 0.5 log_10_ TCID_50_ per mL undiluted, respectively. Both PCV2a and PCV2b challenge material were tested and found to be negative for extraneous agents.

Challenge with virulent PCV2a or virulent PCV2b was conducted 3 weeks post-vaccination (D 21–22). Each pig was inoculated with a total of 4 mL of 1:2 diluted PCV2 challenge virus (PCV2a or PCV2b), 2 mL intranasally (1 mL into each nostril) and 2 mL intramuscularly (in the left neck). Challenge material administered intranasally was applied using a nasal atomizer device (MAD Nasal™ Intranasal Mucosal Atomization Device with Luer-Lock connector). Pigs were observed at 1 h (± 30 min) after challenge for abnormal clinical signs. No abnormal clinical signs were observed following challenge.

#### Necropsy

A general post-mortem examination was conducted by a veterinarian. Tissue samples of three lymph nodes (tracheobronchial, mesenteric, superficial inguinal) and tonsil were collected from each pig and fixed in 10% neutral buffered formalin solution. Sections were stained with hematoxylin and eosin (H&E) and by immunohistochemistry for PCV2 as previously described [28]. Slides were examined for lesions of PCVD: lymphoid depletion (LD) and histiocytic replacement (HR). The same pathologist analyzed all tissues per study; the same pathologist examined the tissues for studies B and C. The amount of PCV2 antigen, LD, and HR in the mentioned tissues were recorded as previously described [29] but with minor modifications: A score of 0 was assigned when LD, HR, and IHC staining were absent; a score of 1 was assigned where < 25% of the tissue architecture was changed (LD or HR) or < 25% of the tissue was IHC positive; a score of 2 was assigned where 25%–75% of the tissue architecture was changed (LD or HR) or 25%–75% of the tissue was IHC positive; a score of 3 was assigned where > 75% of the tissue architecture was changed (LD or HR) or > 75% of the tissue was IHC positive. A score of 0 was considered negative and a score equal or higher to 1 was considered positive. A pig was considered as IHC positive if one or more tissues were IHC positive. A pig was considered to have lymphoid depletion or histiocytic replacement if one or more tissues were abnormal for LD or HR, respectively.

### Study A: experimental design

Study A evaluated the efficacy of a cPCV2a monovalent vaccine in front of challenge with a homologous (PCV2a) and a heterologous (PCV2b) challenge strain. Pigs were randomly assigned to pens and one of four treatment groups, designated T (for treatment) 01–04 per a randomized complete block design. There were 10 uniquely identified pigs per vaccine or treatment control group (Table 1). The control treatment consisted of physiologic saline. The vaccine was an experimental vaccine containing cPCV2a. Two treatment groups (T02 and T04) were inoculated with the experimental cPCV2a vaccine intramuscularly at 23–30 days of age on study day 0. Since this was a pilot study with 10 pigs per group, statistical comparisons could not be made, and summaries were made based on if an animal was “ever positive” for the various efficacy outcomes.

**Table 1:** **Experimental design for study A—homologous vs heterologous protection to a PCV2a Vvaccine**

Treatment (T)	Vaccine or Control	n	Vaccination	Challenge		Necropsy
					Strain	
T01	Saline Control	10	Study Day 0 ^a^ 2 mL intramuscular in right neck	Study Day 21 2 mL intra-muscular in left neck 2 mL intranasal (1 mL each nare)	PCV2a	Study Day 42
T02	cPCV2a	10				
T03	Saline Control	10			PCV2b	
T04	cPCV2a	10				

^a^ Day of study. Pigs were 20–22 days of age on Study Day 0 which corresponded to the day of vaccination.

The efficacy of the vaccine was determined against challenge with PCV2a (T01 and T02) or PCV2b (T03 and T04); the saline vaccinated groups (T01 and T03) were included as challenge controls. Challenge with PCV2a or PCV2b occurred on study day 21, three weeks post-vaccination. At study days -1, 21, 28, 35, and 42, blood and fecal samples were collected for PCV2 DNA detection. On Day 42 animals were euthanized and necropsied.

### Studies B and C: experimental design

Study B and Study C evaluated the potential impact of each cPCV2 vaccine fraction of a bivalent vaccine on the efficacy of the other cPCV2 fraction. Pigs were randomly assigned to pens and one of three treatment groups, designated T01–03 per a randomized complete block design. There were 24 uniquely identified pigs per vaccine or treatment control group (Table 2). In Study B pigs were inoculated with the experimental cPCV2a vaccine (T02), an experimental bivalent PCV2 vaccine containing both cPCV2a and cPCV2b (T03), or physiological saline (T01). In Study C pigs were inoculated with the experimental cPCV2b vaccine (T02), the experimental bivalent PCV2 vaccine containing both cPCV2a and PCV2b (T03), or physiological saline (T01).

**Table 2:** **Experimental design for studies B and C – monovalent and bivalent vaccine evaluation**

Study and Treatment (T)		Vaccine or Control	n	Vaccination	Challenge		Necropsy
						Strain	
Study B	T01	Saline Control	24	Study Day 0 ^a^ 2 mL intramuscular in right neck	Study Day 21 2 mL intramuscular in Left neck 2 mL intranasal (1 mL each nare)	PCV2a	Study Day 42
	T02	cPCV2a	24				
	T03	cPCV2a + cPCV2b	24				
Study C	T01	Saline control	24		Study Day 22 2 mL intramuscular in Left neck 2 mL intranasal (1 mL each nare)	PCV2b	Study Day 43
	T02	cPCV2b	24				
	T03	cPCV2a + cPCV2b	24				

^a^ Day of study. Pigs were 20–22 days of age on Study Day 0 which corresponded to the day of vaccination.

The tested vaccine serials and control product were administered intramuscularly once to pigs at 20–24 days of age (on study day 0) per designated treatment group (T01–T03). The efficacy of each serial was determined against PCV2a challenge in Study B and PCV2b challenge in Study C, administered 21–22 days post-vaccination. At study days 21, 24, 28 or 29, 31, 35, 38, and 42 or 43, blood and fecal samples were collected for PCV2 DNA or antibody detection. On study day 42 or 43 animals were euthanized and necropsied.

Study B and Study C were conducted as two separate studies to obtain best statistical models possible. There were minor differences in sample collection days among studies B and C. Statistics were performed among treatment groups (T01–T03) within each study.

### Efficacy outcome criteria

Vaccination was considered effective as an aid in the prevention of PCV2 viremia if a significant (*P* ≤ 0.05) reduction of post-challenge PCV2 viremia and/or percent ever different from sham vaccinated animals was achieved in vaccinated groups T02 and T03 compared to controls (T01) for Studies B and C. For Study A values were summarized as study groups were too small for statistical analysis. Four pigs from Study A, 9 pigs from Study B and 1 pig from Study C were removed from the respective studies for different health reasons. The data from these animals were not included in the analysis of results.

#### PCV2 DNA quantification in serum and fecal swab samples

DNA was extracted from serum and fecal swab samples using a commercial kit (QiaAmp Blood 96 kit, Qiagen) and real-time quantitative PCR (qPCR) was performed. Five μL of DNA were used as template, reverse transcribed at 50 °C for 2 min, and denatured at 95 °C for 10 min. The PCR program consisted of 40 cycles of denaturation at 95 °C for 25 s and annealing/extension at 60 °C for 1 min. The RT-qPCR was conducted in a thermocycler (Bio-Rad CFX 96). The sequences of primers and probe were as described [30]. The number of DNA copies obtained are expressed as number of copies per 5 μL.

#### Viremia and fecal shedding

Post-challenge qualitative (frequency of ever viremic) and quantitative (amount of PCV2 in serum samples) viremia were the primary outcome variables. Qualitative (frequency of ever having fecal shedding) and quantitative (amount of PCV2 in fecal samples) PCV2 virus shedding post-challenge in fecal samples was another outcome variable. Frequency distributions of viremia and fecal shedding were calculated for each treatment and post-challenge time point data were collected. It was determined if an animal was ever viremic or ever shed PCV2 in its feces post-challenge. The qPCR data (copy number) were transformed with an appropriate log transformation prior to analysis. The transformed data were analyzed with a general linear repeated measures mixed model. The model included the fixed effects of treatment, time point, and treatment by time point interaction and the random effects of pen, block within pen, and treatment by block within pen (which is the animal term). The generalized linear mixed model did not converge, so a Cochran-Armitage test adjusting for pen was used to analyze the data. Contrasts comparing T01 to T02 and to T03 were made in studies B and C. Least squares means (back-transformed), standard errors (back-transformed), 95% confidence intervals of the means (back transformed) and ranges were calculated for each treatment at each time point.

##### ELISA

PCV2 antibody values (SERELISA® PCV2 Ab Mono Blocking enzyme linked immunosorbent assay (ELISA; performed per manufacturer instructions) were transformed with an appropriate log transformation prior to analyses Studies B and C). Transformed values were analyzed with a general linear repeated measures mixed model. The model included the fixed effects of treatment, time point, and treatment by time point interaction and the random effects of pen, block within pen, and treatment by block within pen interaction (which is the animal term). Comparisons were made between treatment T01 and treatments T02-T03 at each time point using contrasts. Least squares means (back-transformed), standard errors, 95% confidence intervals of the means, ranges were calculated for each treatment at each time point.

#### Lymphoid depletion and histiocytic replacement

Frequency distributions were calculated from microscopic lesion scores [lymphoid depletion (LD)] for each tissue assayed by treatment group. It was determined if an animal was abnormal for either LD or histiocytic replacement (HR). Frequency distributions of normal/abnormal results were calculated for each treatment for LD and HR separately as well as either LD and/or HR. Normal/abnormal results for LD and LD and/or HR were analyzed with a generalized linear mixed model with the binomial distribution and logit link function. The model contained the fixed effect of treatment and random effects of pen and block within pen. A Cochran-Armitage test adjusting for pen was used to analyze the data for HR since the generalized linear mixed model did not converge. Contrasts were used to compare treatment T01 to treatments T02 and T03. For LD and LD and/or HR normal/abnormal results, the generalized linear mixed model converged, so back-transformed least squares means, standard errors, and 95% confidence intervals of the means were calculated for each treatment.

#### Virus infection in lymphoid tissues (IHC)

An animal was considered positive for PCV2 colonization if any tissue was positive [28, 29]. IHC was used to confirm virus localization within cells. Normal/abnormal results were analyzed with a generalized linear mixed model with fixed effect treatment and random effects pen and block within pen. The generalized linear mixed model used the binomial distribution and logit link function. Comparisons were made between treatment T01 and treatments T02 and T03 using contrasts. Back-transformed least squares means, standard error, and 95% confidence intervals of the means were calculated for each treatment.

### T cell epitope content comparison (EpiCC) analysis

Since biologically differential efficacy was observed, T cell epitope content comparison (EpiCC) analysis was performed to quantify the relatedness of the cPCV2a and cPCV2b vaccine fractions to the PCV2a and PCV2b challenge. The EpiCC analysis was conducted as previously described [24]. T cell epitopes were identified based on predicted binding to swine leukocyte antigen (SLA) alleles, including eight MHC class I alleles (SLA‐1*0801, 1*1201, 1*1301, 2*0501, 2*1201, 3*0501, 3*0601, and 3*0701) and five SLA class II alleles (SLA‐DRB1*0201, 0402, 0602, 0701, and 1001). The 9-mer peptides that scored in the top 5% of predicted binding were used for the EpiCC analysis. The EpiCC algorithm, compares two protein sequences and renders results as an EpiCC score which quantifies the relatedness of the putative epitope content shared between a given pair of sequences (for example vaccine versus challenge). More similar shared epitope content between the vaccine and field strain sequences results in a greater EpiCC score. Each sequence was compared to itself to determine its baseline EpiCC score, which represents the T cell epitope content of that sequence. To quantify vaccine T cell epitope coverage, the EpiCC score of each challenge strain comparison was divided by that challenge strain baseline EpiCC score and expressed as a percentage.

## Results

### Study validity

Study animals did not have signs of infection with PCV2, or other infectious agents, that may have impacted viremia, fecal shedding, or lymphoid colonization, lymphoid depletion, or histiocytic replacement in lymphoid tissues in this model. All pigs were negative for PCV2 antibodies (SERELISA S/N ratio > 0.5) prior to vaccine or saline administration and the control groups (T01) remained negative for PCV2 antibodies (SERELISA S/N ratio > 0.5) until post-challenge.

### Study A outcomes

Challenge with pathogenic PCV2a and PCV2b induced viremia, fecal shedding, and histological lesions in control animals (Groups T01 and T03, respectively). In T01 PCV2a challenge resulted in viremia and fecal shedding which were both first detected on study day 28 (Table 3). Peak viremia (22 982 DNA copies/5 µL) and fecal shedding (23 485 DNA copies/5 µL) in T01 animals occurred on Day 35 and 42 of the study, respectively. The percent of control animals with abnormal histopathological lesions ranged from 20 to 70% depending on the specific outcome. cPCV2a vaccinated and PCV2a challenged animals (T02) were first viremic on study day 28 and this was also the day of peak viremia (43 DNA copies/5 µL). T02 animals first shed PCV2 in their feces on study day 35 and this was also the day of peak fecal shedding (16 DNA copies/5 µL). In T03 animals PCV2b challenge resulted in viremia and fecal shedding which were both first detected on study day 28. Peak viremia (280 798 DNA copies/5 µL) and fecal shed (106 798) in T03 animals occurred on Day 35 and 42 of the study, respectively. Further the percent of control animals with abnormal histopathological lesions ranged from 66.7 to 77.8% depending on the specific outcome. cPCV2a vaccinated and PCV2b challenged animals (T04) were first viremic on study day 28 and this was also the day of peak viremia (1866 DNA copies/5 µL). T04 animals first shed PCV2 in their feces on study day 28; study days 28 and 35 were the days of peak fecal shedding (7 DNA copies/5 µL). The PCV2a vaccine reduced the peak values and number of animals ever positive for viremia, fecal shedding, lymphoid lesions, and lymphoid colonization following challenge with either PCV2a or PCV2b compared to sham vaccinated controls (Table 4). The homologous vaccine-challenge group (T02) resulted in biologically improved efficacy outcomes compared to heterologous vaccine-challenge group (T04).

**Table 3:** **Summary of back-transformed LS means PCV2 copies/5 µL in serum (viremia) or feces (fecal shedding) by qPCR by treatment and study day for study A**

Treatment Group (T) and description			Endpoint	n	Day of study				
	Vaccine	Challenge			− 1	21	28	35	42
T01	Saline	PCV2a	Viremia	10	0	0	1.09E + 04	2.30E + 04	1.91E + 03
T02	cPCV2a	PCV2a		10	0	0	43	2	2
T03	Saline	PCV2b		9	0	0	3.04E + 04	2.81E + 05	5.18E + 03
T04	cPCV2a	PCV2b		10	0	0	1.87E + 03	22	2
T01	Saline	PCV2a	Fecal Shedding	10	0	0	1.84E + 02	4.13E + 03	2.35E + 04
T02	cPCV2a	PCV2a		10	0	0	0	16	2
T03	Saline	PCV2b		9	0	0	2.57E + 02	2.48E + 04	1.07E + 05
T04	cPCV2a	PCV2b		10	0	0	7	7	0

Study Day 0 corresponded to the day of vaccination.

^*^ One animal from T03 and one animal from T04 were removed for health reasons.

**Table 4:** **Summary of study A results—homologous vs heterologous protection to a PCV2a vaccine**

Protection Endpoint	Treatment Group (T)			
	T01—PCV2a challenge control	T02—cPCV2a Vaccine	T03—PCV2b challenge control*	T04—cPCV2a Vaccine*
	PCV2a challenge (homologous)		PCV2b challenge (heterologous)	
	Ever positive positive #/total # (%)			
Viremia	9/10 (90%)	5/10 (50%)	9/9 (100%)	7/10 (70%)
Fecal Shedding	9/10 (90%)	3/10 (30%)	9/9 (100%)	3/9 (33.3%)
Lymphoid Depletion (LD)	6/10 (60%)	0/10 (0%)	6/9 (66.7%)	4/9 (44.4%)
Histiocytic Replacement (HR)	2/10 (20%)	0/10 (0%)	6/9 (66.7%)	2/9 (22.2%)
Lymphoid Colonization (IHC)	7/10 (70%)	4/10 (40%)	7/9 (77.8%)	3/9 (33.3%)

^*^ One animal from T03 and one animal from T04 were removed for health reasons.

### Study B and C outcomes

#### PCV2 viremia

In both studies B and C, animals were negative for PCV2 viremia prior to challenge. Day 28 or 29 was the first day viremia was detected in Study B and Day 24 was the first day viremia was detected in Study C (Table 5). Starting on Day 28 or 29 and continuing for the remainder of the study, vaccinated groups T02 and T03 had significantly lower (*P* < 0.0001 for Study B, *P* ≤ 0.0017 for Study C) PCV2 viremia compared to T01. For Study B PCV2 viremia in T01 animals peaked on Day 35 (13 days post-challenge) and continued through Day 42. Some vaccinated animals (T02 and T03) were PCV2 viremia positive from Day 28 or 29; however, only 36.4% and 26.1% of T02 and T03 animals, respectively, were ever viremia positive compared to 100% in T01 controls (*P* < 0.0001 for both vaccinate groups compared to control). For Study C PCV2 viremia in T01 animals peaked at day 31 (11 days post-challenge) and continued through day 42. One T03 animal was viremia positive on Day 24; T02 first became viremia positive on Day 28 or 29. While 100% of T01 animals were viremia positive, 52.4% and 40.9% of T02 and T03 animals, respectively (*P* ≤ 0.05 for both vaccinate groups compared to control) were ever viremia positive.

**Table 5:** **Summary of back-transformed LS means PCV2 copies/5 µL in serum (viremia) by treatment group and study day for studies B and C (evaluation of monovalent vs bivalent vaccines)**

Study	Treatment Group (T)	Day of study								% pigs ever positive post challenge
		− 1	21	24	28 or 29	31	35	38	42	
B	T01	0	0	0	5.69E + 03	1.72E + 04	1.84E + 04	1.38E + 04	1.44E + 04	100
B	T02	0	0	0	0.6*	2.0*	4.4*	5.9*	2.9*	36.4*
B	T03	0	0	0	1.4*	1.4*	0.5*	0.7*	0.5*	26.1*
C	T01	0	0	0	7.34E + 02	1.76E + 04	9.68E + 02	1.22E + 04	2.33E + 02	100
C	T02	0	0	0	13.5**	21.8**	1.8**	5.6**	0.8**	52.4**
C	T03	0	0	0.6	4.5**	1.4**	0.6**	5.7**	0.5**	40.9**

Value is significantly different from T01 saline controls (*P < 0.0001, **P ≤ 0.05) within a column (day of study; study day 0 corresponded to the day of vaccination).

#### PCV2 fecal shedding

T02 and T03 vaccinated groups had a significantly lower (*P* ≤ 0.03) percentage of animals that ever shed PCV2 in feces post-challenge compared to T01 for Study B (Table 6). The percent of animals ever fecal shedding in Study C were not statistically different from saline control group. On Day 29 and all subsequent sampling days, the vaccinated groups (T02 and T03) had significantly lower levels of fecal shedding compared to T01 for Study B and C (Table 6).

**Table 6:** **Summary of back-transformed LS means PCV2 copies/5 µL in feces (fecal shedding) by qPCR by treatment and study day for studies B and C**

Study	Treatment Group (T)	Day of Study								% pigs ever positive post-challenge
		− 1	21	24	28 or 29	31	35	38	42	
B	T01	0	0	7	2.01E + 02	6.45E + 03	2.46E + 04	2.28E + 04	9.62E + 03	100
B	T02	0	0	14.0	1.0*	14.0*	8.0*	3.0*	11.0*	68.2**
B	T03	0	0	7.0	0*	1.0*	12.0*	4.0*	1.0*	47.8**
C	T01	0	0	2.0	26	9.99E + 04	1.95E + 04	3.47E + 03	7.95E + 03	100
C	T02	0	0	1.0	5.0	1.33E + 03**	2.20E + 02**	1.40E + 02**	14.0**	90.5^
C	T03	0	0	1.0	3.0	3.71E + 02**	2.15E + 02**	5.30E + 02**	14.0**	86.4^

Value is significantly different from T01 saline controls (*P ≤ 0.0003, ** *P* ≤ 0.05) within a column (Day of Study) by Study. ^Value is not significantly different from T01 (*P* ≥ 0.1). Study Day 0 corresponded to the day of vaccination.

#### Serology

On study day -1 and day 21, all groups (T01, T02 and T03) were negative for PCV2 antibodies for both Studies B and C (Table 7). There were significant differences identified between the vaccinated group T03 compared to T01 on Days 21 and 24 for Study B and on Day 21 for Study C, but these values are negative, based on the assay positive/negative cut-off level. On Days 29 to 42 for Study B and Day 42 for Study C, T02 and T03 vaccinated groups were positive for PCV2 antibodies based on SERELISA values and had significantly higher PCV2 antibody levels compared to T01 (Table 7). Anamnestic serologic responses were detected in all vaccine groups post-challenge.

**Table 7:** **Back-transformed LS means PCV2 SERELISA values by treatment and study day for studies B and C**

Study	Treatment Group (T)	Day of Study							
		− 1	21	24	28 or 29	31	35	38	42
B	T01	0.809	0.884	0.839	0.882	0.842	0.598	0.605	0.560
B	T02	0.808	0.852	0.804	0.478*	0.357*	0.203*	0.196*	0.158*
B	T03	0.805	0.733*	0.722*	0.329*	0.290*	0.186*	0.160*	0.130*
C	T01	0.844	0.877	ND	ND	ND	ND	ND	0.479
C	T02	0.867	0.784*	ND	ND	ND	ND	ND	0.202**
C	T03	0.862	0.709*	ND	ND	ND	ND	ND	0.136**

Value is significantly different from T01 saline controls (*P ≤ 0.0013, ** *P* ≤ 0.05) within the column (Day of Study; Study Day 0 corresponded to the day of vaccination). All values on Days − 1 and 21 are considered negative based on the assay positive/negative cut-off. SERELISA: the lower the S/Nc SERELISA value the more positive the value is and the higher the antibody level.

ND: not determined.

#### Lymphoid depletion, histiocytic replacement, and virus infection (colonization) in lymphoid tissues (immunohistochemistry)

In both studies some animals in all treatment groups were positive for LD and/or HR. T02 and T03 vaccinated groups had significantly less LD and/or HR compared to T01 (Table 8). In both studies some animals in all treatment groups were positive for PCV2 colonization of lymphoid tissues. For Study B, T02 and T03 vaccinated groups had significantly (*P* ≤ 0.0004) fewer animals positive for PCV2 colonization by IHC compared to T01. For Study C both T02 and T03 had significantly (*P* ≤ 0.05) fewer animals positive by IHC compared to saline controls.

**Table 8:** **Back-transformed LS means for if ever positive for PCV2 colonized lymph tissues (immunohistochemistry) and lymphoid depletion and/or histiocytic replacement by treatment for studies B and C**

Study	Treatment Group (T)	PCV2 colonized lymph tissues (immunohistochemistry) ^a^	Lymphoid depletion and/or histiocytic replacement ^a^
B	T01	95.5 (72.1–99.4)	90.9 (69.1–97.8)
B	T02	18.8 (6.5–43.7) *	14.3 (4.5–37.0) **
B	T03	8.8 (2.0–31.6) *	4.5 (0.6–27.4) **
C	T01	96.0 (69.0–99.6)	86.2 (60.6–96.2)
C	T02	56.3 (23.5–43.7) ***	37.2 (16.9–63.4) ***
C	T03	35.2 (11.3–31.6) ***	28.1 (11.0–55.3) ***

^a^ Back-transformed least squares means (back transformed Lower 95% CI—back transformed upper 95% CI).

^*^Value is significantly different from T01 saline controls (*P ≤ 0.0004, ** *P* ≤ 0.0001, *** *P* ≤ 0.05).

### Outcome summary

In both studies B and C, the animals vaccinated with the bivalent (cPCV2a–cPCV2b) vaccine were biologically better protected from PCV2 infection and disease than animals vaccinated with a monovalent cPCV2a or cPCV2b vaccine (Table 9). Following PCV2a challenge, the cPCV2a–cPCV2b vaccine offered a biologically relevant decrease in the frequency of viremia (28.3%) compared to the monovalent PCV2a vaccine. Following PCV2b challenge, the cPCV2a-cPCV2b vaccine offered a biologically relevant decrease in the frequency of PCV2 viremia (21.9%), compared to the monovalent cPCV2b vaccine. The decrease in percentage of bivalent vaccinated pigs that were ever viremic post-challenge was significantly lower than monovalent vaccinated pigs (*P* < 0.0001) in both Study B and Study C. Similarly, fecal shedding, LD, LD or HR, lymphoid colonization, and serologic outcomes were improved in animals receiving the bivalent vaccine versus monovalent vaccines in both studies B and C.

**Table 9:** **Study B and Study C– Summary of efficacy outcomes imparted by the bivalent vaccine compared to monovalent vaccines for Studies B and C**

Protection Endpoint	% Improvement of PCV2a-PCV2b bivalent vs:	
	PCV2a monovalent^2^	PCV2b monovalent^2^
Viremia	28.3%	21.9%
Fecal Shedding	29.9%	4.5%
Lymphoid Depletion (LD)^1^	68.5%	25.1%
Histiocytic Replacement (HR) ^1^	NA	100%
LD or HR^1^	68.5%	24.5%
Lymphoid Colonization (IHC) ^1^	53.3%	37.5%
Serology^1*^	17.7%	32.7%

^1^ Presented as back-transformed least square means.

^*^The ELISA used to determine PCV2 specific antibodies (serology) was an S/N (sample to negative ratio) ELISA where seropositive < 0.5. Serology data noted are from a blood sample collected at necropsy.

^2^ The percent improvement of the bivalent vaccine over the monovalent vaccine has been calculated for each outcome criteria ((monovalent-bivalent)/monovalent).

### T cell epitope content comparison (EpiCC)

In the situation where the vaccine and challenge strain were both PCV2a, the vaccine offered 100% epitope coverage (Table 10). On the other hand, in the situation where the vaccine and challenge strain were mismatched, the PCV2a vaccine offered 72.05% coverage to the PCV2b challenge.

**Table 10:** **T cell epitope content comparison (EpiCC) analysis**

Treatment Group (T) and description			EpiCC scores		Percent coverage		% Improvement of homologous vs heterologous coverage ^a^
	Vaccine	Challenge	PCV2a ORF2 ^b^	PCV2b ORF2 ^c^	PCV2a ORF2 ^b^	PCV2b ORF2 ^c^	
T02 homologous treatment	cPCV2a	PCV2a	10.42 ^d^	7.51	100.00	72.05	36.46
T04 heterologous treatment	cPCV2a	PCV2b	7.51	10.25 ^d^	73.28	100.00	

^a^ The percent improvement of homologous (cPCV2a vaccine, PCV2a challenge) versus heterologous (cPCV2a vaccine, PCV2b challenge) coverage (|(heterologous-homologous)|/heterologous * 100%) representing the situation in Study A.

^b^ Situation tested in studies A and B.

^c^ Situation tested in study C.

^d^ Represents baseline where the challenge strain is identical to the vaccine strain.

## Discussion

There is a growing need for an improved PCV2 swine vaccine and a strategy to match vaccines to field genotypes as genotypes evolve. PCV2a based vaccines have been highly successful in controlling PCV2a viral infections, however, widespread use of PCV2a vaccines has contributed to the selection of vaccine-immune resistant PCV2 mutants/genotypes. For PCV2a, directional selection appears to have induced a change in the viral capsid away from the vaccine specific antigenic determinants (especially regarding capsid epitopes) [5]. When immunity is not sterilizing wild-type strains are able to circulate in a population of less susceptible hosts. This immune-escape phenomenon has been recognized for Hepatitis B virus, Avian metapneumovirus, an increase in virulence in avian Marek’s disease, or both for avian Infectious Bursal Disease [21]. If vaccination has an effect on viral evolution and/or selection of different strains, it can impact viral populations including emergence of new viral genotypes in host populations, increase selective pressure especially on epitope (PCV2 capsid) regions, and promote a tendency of viruses to evolve in a way to distance away from vaccine strains’ genomic sequences [5]. Taken together, there are increasingly more PCV2 strains, including recombinants, and genotypes that may escape from traditional PCV2a-vaccine induced immunity.

A review [18] summarized several research studies involving controlled lab and field studies providing non-biased evidence that PCV2 monovalent vaccines provide good homologous but less heterologous protection to varied PCV2 genotypes. Based on the studies highlighted in the review [18], PCV2 vaccines were described as “leaky”; PCV2 vaccines are often able to induce protection against clinical disease but not stop infection or viral transmission. Study A of the current investigation explored the ability of a cPCV2a vaccine to offer homologous (matched PCV2 genotype challenge) and heterologous (non-matched genotype challenge) protection. The cPCV2a vaccine offered biologically relevant enhanced protection against PCV2a challenge compared to PCV2b challenge for viremia, fecal shedding, and lymphoid depletion and histiocytic replacement. Lymphoid colonization was the only endpoint for which homologous vaccine-challenge was not biologically improved over heterologous vaccine-challenge and this difference may be an artifact of the group size or outcome difference in one animal. It is also possible the PCV2b virus is more virulent than the PCV2a challenge virus; while direct comparisons cannot be made across independent studies, there were no obvious differences in the clinical presentation of PCV2a or PCV2b challenged control animals. In summary, protection imparted from a PCV2 vaccine is likely best when the vaccine and challenge strains were closely matched.

Results of Study A prompted an EpiCC analysis to understand if the relatedness of T cell epitopes among the cPCV2a vaccine and PCV2a and PCV2b challenge strains might be contributing to biologically differential protective immunity. PCV2 vaccine-induced protection to challenge viruses can be at least partially attributed to the epitopic determinants common or unique among those viruses [11, 15–17]. EpiCC analysis confirmed the cPCV2a vaccine and PCV2a challenge strain were 100% related (as expected), and only some of the epitopes in the cPCV2a vaccine and PCV2b challenge were related (73.28%). The cPCV2a vaccine offered 36.46% greater coverage to PCV2a challenge than to PCV2b challenge and this increased breadth of coverage is consistent with the improvement in protective efficacy offered by the cPCV2a vaccine to PCV2a challenge compared to PCV2b challenge. Taken together with the clinical results of Study A, EpiCC results may be practically informative of clinical vaccine-induced protection. Therefore, results from Study A support the justification to test a bivalent vaccine to enhance protection of pigs and as a means to enhance the breadth of epitope overlap among vaccine and diverse challenge strains.

Two major phylogenic groups of PCV2 exist, namely the PCV2a and PCV2b/d genogroups [31]. This classification of genogroups is supported on an epitope level in the higher degree of shared epitopes among PCV2b and PCV2d compared to PCV2a and PCV2d [15–17]. Combining cPCV2b and cPCV2a vaccine strains into one vaccine provides a representative vaccine virus for each of the major PCV2 groups and expands the epitopes included in the vaccine. In Studies B and C, animals vaccinated with the cPCV2a–cPCV2b bivalent vaccine were protected from PCV2 infection and disease. Animals treated with the bivalent vaccine shed numerically less PCV2 in their feces and had numerically less PCV2 in their blood compared to animals treated with the monovalent vaccine. Further, there were numerically less bivalent-vaccine treated animals that were ever viremic or ever shed PCV2 in their feces compared to monovalent vaccine treated animals. These results are consistent with results of a study focused on determination of the relatedness of PCV2 vaccine and field strains in terms of T cell epitopes [31]. The baseline percent coverage offered by the bivalent cPCV2a–cPCV2b vaccine is 100% to each challenge strain. In this way, the cPCV2a–cPCV2b bivalent vaccine potentially offers at least the same efficacy as each vaccine fraction individually. Yet, in studies B and C, the benefit of the bivalent vaccine was greater than just the shared epitopes among the vaccine and field strains. The directional enhancement in efficacy offered by the bivalent vaccine compared to each monovalent may be due to the availability of epitopes to interact with the immune system and induce a broad repertoire of responding T lymphocytes. Expanding the epitopes in the vaccine should broaden the ability of pigs to respond appropriately to diverse PCV2 field strains. Studies with higher power or use of more divergent challenge strains is needed to further define and elucidates the immune bases for the enhanced clinical protection. Nonetheless, this work supports the need, and high probability of success, for updating current monovalent PCV2 vaccines to include multiple PCV2 genotypes.

The selection of vaccine genotypes should consider not only the T cell epitope content but also the likelihood of inducing T memory cells that will recognize epitopes in PCV2 viruses circulating in the swine population. In this way vaccines would be able to induce immune responses that would also match T cell epitopes present in field genotypes. Bandrick et al. [31] performed T cell epitope content comparison to evaluate the T cell epitope relatedness between PCV2 vaccine and field genotypes. T cell epitopes predicted to bind class I and II swine leukocyte antigen (SLA) alleles to capsid (ORF2 encoded) and replicase (ORF1 encoded) proteins were evaluated for relatedness in: A) 2 commercial PCV2a vaccines (including a cPCV2a vaccine), an experimental cPCV2b vaccine, and an experimental cPCV2a-cPCV2b vaccine, and B) 161 PCV2 field isolates obtained from GenBank including genotypes a-f that were found in field clinical disease cases. In general, PCV2a vaccines had higher scores (greater relatedness) to PCV2a field genotypes. The experimental cPCV2b vaccine had good relatedness to both PCV2b and PCV2d field virus sequences. The in-silico results [31] agreed with the in vivo results observed in this investigation. This study demonstrates a correlation among EpiCC score, percent coverage, and clinical protection from PCV2 infection and disease. Furthermore, it has been demonstrated that the cPCV2a–cPCV2b vaccine induces cell-mediated immunity (CMI) (as measured by PCV2-specific IFN-γ production) following one or two doses [32]. Taken together, the cPCV2a–cPCV2b vaccine’s greater T cell epitope overlap to field strains, its ability to induce robust CMI responses, as well as its enhanced protective efficacy in the face of PCV2a and PCV2b challenge, should greatly increase the breadth of protection in the field. A study evaluating clinical protection imparted by the bivalent or monovalent vaccine to a divergent PCV2 challenge virus is a potential opportunity to test this hypothesis.

PCV2 will continue to evolve and by many mechanisms. Continuous monitoring for new PCV2 genotypes and understanding of the molecular/biological importance of new mutations will help identify the potential emergence of vaccine resistant strains and to update vaccines to match epitopes of vaccines to wild type viruses [5, 12]. Importantly, PCV2 bivalent vaccines may impact selection pressure that adversely affects the ability of circulating PCV2 strains and their mutant derivatives to propagate [33–38]. Updating traditional PCV2 vaccines to include multiple genotypes may induce broader and more complete protection, as well as potentially slow the evolution, of the diverse and changing PCV2 virus.

Providing a bivalent cPCV2a–cPCV2b vaccine offers superior protection compared to monovalent vaccines against heterologous PCV2 because of epitopic overlap to diverse strains and potentially because of enhanced availability of epitopes to the immune system. Broader antigenic coverage offered by the inclusion of two PCV2 genotypes will be expected to greatly benefit the swine industry by increasing the breadth of vaccine-induced protection. This series of studies support the need, and suggest a high probability of success, for updating current monovalent PCV2 vaccines to include other genotypes. This will broaden the immune status of pigs and with positive implications to reduce the rate of emergence of resistant strains of PCV2.
